# Functional and metabolomic analyses of brown adipose tissue during cold-deacclimation reveal rapid N-acetylated amino acid adaptations

**DOI:** 10.1016/j.isci.2026.115146

**Published:** 2026-02-25

**Authors:** Chantal A. Pileggi, Ella McIlroy, Lauren M.K. Hamilton, Nidhi Kuksal, Luke S. Kennedy, Valeria Vasilyeva, Michel N. Kanaan, Ziyad El Hankouri, Yan Burelle, Miroslava Cuperlovic-Culf, Mary-Ellen Harper

**Affiliations:** 1 Department of Biochemistry, Microbiology, and Immunology, Faculty of Medicine, University of Ottawa, Ottawa, ON, Canada; 2 Ottawa Institute of Systems Biology, University of Ottawa, Ottawa, ON, Canada; 3 Interdisciplinary School of Health Sciences, Faculty of Health Science, University of Ottawa, Ottawa, ON, Canada; 4 Department of Cellular and Molecular Medicine, Faculty of Medicine, University of Ottawa, Ottawa, ON, Canada; 5 National Research Council of Canada, Digital Technologies Research Centre, 1200 Montreal Road, Ottawa, ON K1A 0R6, Canada

**Keywords:** physiology

## Abstract

Non-shivering thermogenesis (NST) in brown adipose tissue (BAT) is rapidly activated in the cold but inactive at warm ambient temperatures. To elucidate the metabolic remodeling in BAT during recovery from cold exposure, mice were acclimated to 4°C for 7 days, then deacclimated at thermoneutrality (30°C) for 3–48 h. Cold-acclimated mice demonstrated high metabolic rates and food intake, which decreased immediately by ∼40% upon deacclimation. Uncoupled respiration decreased by 24 h, corresponding with gradual declines in mitochondrial protein content and UCP1 gene expression. Decreases in BAT mitochondrial content paralleled declines in protein content by 48 h of cold deacclimation. Metabolomic profiling revealed major alterations in amino acid, TCA cycle, glutathione, and purine metabolism pathways. Marked decreases in the abundance of N-acetylated amino acids in cold deacclimated mice corresponded with increased aminoacylase 1 (Acy1) expression. Together, results highlight the coordinated structural and metabolic remodeling of BAT mitochondria during thermogenesis and deactivation.

## Highlights

• Cold-to-thermoneutral transition rapidly decreases whole-body energy expenditure
• BAT mitochondrial content and uncoupling capacity regress in parallel
• Cold-deacclimation decreases succinate and increases purine nucleotides levels
• N-acetylated amino acids may act as valuable indicators of brown adipose thermogenesis

## Graphical abstract

**Figure:**
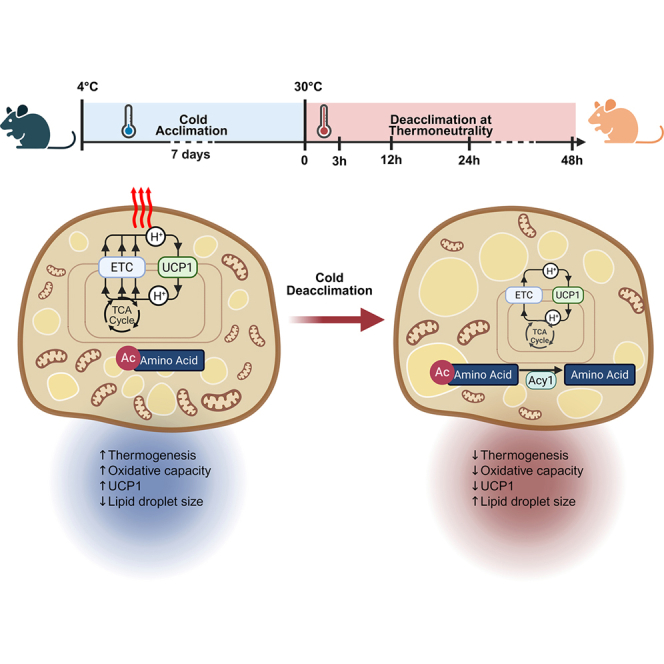

## Introduction

Brown adipose tissue (BAT) serves as an important thermoregulatory organ in homeothermic mammals, functioning to release energy as heat in cold environments through the process of non-shivering thermogenesis (NST).^1^^,^^2^^,^^3^ BAT is highly innervated and vascularized and is abundant in mitochondria that express high levels of the mitochondrial inner membrane protein, uncoupling protein 1 (UCP1).^4^^,^^5^ UCP1 mediates proton conductance across the inner mitochondrial membrane bypassing ATP synthase, thereby dissipating proton motive force and increasing substrate oxidation, which results in thermogenesis.^6^^,^^7^

The molecular events involved in the initiation of cold-induced NST in small mammals are well-defined: exposure to low ambient temperatures stimulates sensory thermal receptors in the skin to activate the sympathetic nervous system. The release of catecholamines, such as norepinephrine, stimulates β-adrenergic receptors on mature brown adipocytes.^8^^,^^9^ Stimulation of β-adrenergic receptors increases cAMP concentrations and activates protein kinase A (PKA), which promotes lipolysis of triglycerides. Mobilized FFAs activate UCP1-mediated proton leak, leading to the increased uptake and rapid oxidation of fatty acids and glucose, which overall results in increased whole-body energy expenditure. Liberated fatty acids also stimulate PGC1α-mediated mitochondrial biogenesis and increase UCP1 expression.^6^^,^^10^^,^^11^ Activation of the hypothalamic-pituitary-thyroid axis increases the supply of thyroid hormone to BAT. As well, norepinephrine-induced increases in brown adipocyte cAMP levels stimulate the conversion of thyroxine (T4) to 3,5,3′-triiodothyronine (T3) via the type 2 iodothyronine deiodinase (DIO2).^12^^,^^13^^,^^14^^,^^15^ Within mature brown adipocytes, mobilized FFAs activate UCP1-mediated proton leak, leading to the increased uptake and rapid oxidation of fatty acids and glucose. In rodent studies, sustained or repeated bouts of cold exposure over periods of >5–7 days is termed cold acclimation, with full adaptation to the cold occurring after ∼3 weeks.^16^ Cold acclimation results in an adapted metabolic state which includes increased vascularization and sympathetic innervation to support brown adipocyte proliferation and differentiation underlying BAT hyperplasia and hypertrophy.^17^^,^^18^^,^^19^ Rapid increases in BAT metabolic activity precede the molecular remodeling of brown adipocytes during cold-acclimation, consistent with the idea that certain metabolites, such as N-acetylamino acids, may function as signaling molecules that not only support acute thermogenic responses but also drive molecular remodeling of the tissue to support increased thermogenic capacity.^20^^,^^21^

Despite the consensus that BAT is inactive under warm ambient environments, few studies have sought to examine the molecular events and metabolic remodeling that occur when recovering from the cold and re-acclimating to thermoneutral environments (28°C–32°C for mice).^22^^,^^23^ Understanding the profound metabolic remodeling that leads to the restitution of active thermogenic BAT into inactivated BAT is crucial for a comprehensive understanding of adaptive thermogenesis processes. Excessive or inappropriate cessation of BAT thermogenesis could potentially lead to hypothermia in humans, for example, in neonates, who have much higher levels of BAT than adults. Elucidating the mechanisms through which BAT thermogenesis is switched off may provide insights into novel ways to activate BAT thermogenesis in certain metabolic diseases for their prevention or treatment. Here, we present a time-resolved analysis of the metabolic and structural remodeling that occurs during BAT inactivation in response to cold deacclimation. We hypothesized that cold deacclimation would result in remodeling that corresponds with rapid decreases in BAT mitochondrial content and oxidative capacity. Our results demonstrate that rapid changes in whole-body metabolism in response to cold deacclimation precede the molecular remodeling of BAT mitochondria and suggest that N-acetylated amino acids correlate with the cessation of thermogenesis and may function as metabolite biomarkers in BAT.

## Results

### Resting metabolic rate rapidly decreases during cold-deacclimation at thermoneutrality

**Figure 1:**
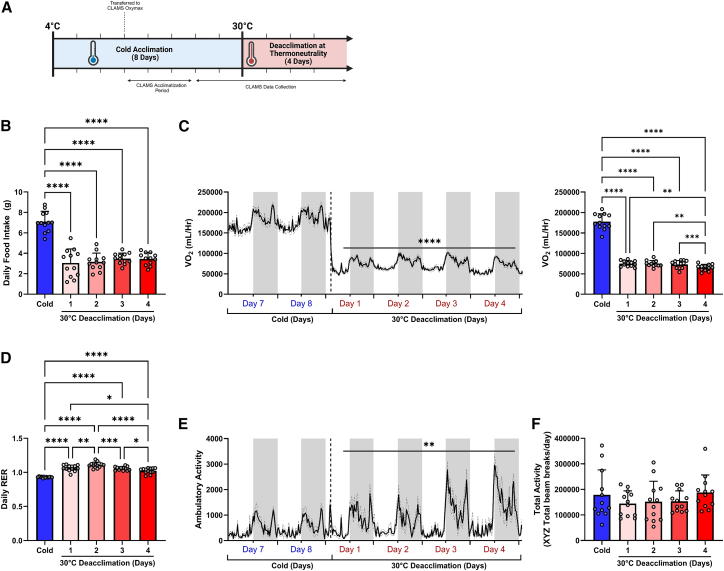
Resting metabolic rate rapidly decreases during cold-deacclimation (A–F) Comprehensive metabolic phenotyping analyses were measured using a fully automated CLAMS system (Columbus Instruments) during cold acclimation (4°C) and subsequent deacclimation at thermoneutrality (30°C). (A) Schematic summary of the time points for sample collection. Diagram created with BioRender.com. (B) Daily *ad libitum* cumulative food intake. (C) Average hourly metabolic rates (VO2). (D) Respiratory exchange ratio (RER; VCO2/VO2). (E) Ambulatory activity was calculated as the sum of ambulatory beam breaks in the x, y, and z planes. (F) Total activity was calculated as the sum of all beam breaks in the x, y, and z planes. Comparisons between time points were determined using a one-way RM ANOVA with Tukey post-hoc tests, ∗*p* < 0.05, ∗∗*p* < 0.01, ∗∗∗*p* < 0.001, ∗∗∗∗*p* < 0.0001. All values are presented as means ± SD.

To assess the metabolic adaptations of cold deacclimation, C57BL/6J mice were acclimated to the cold (4°C for 8 days) and then transferred to a thermoneutral climate (30°C) for 4 days (Figure 1A). Consistent with previous findings,^6^^,^^24^ *ad libitum* food intake was high during cold acclimation and immediately decreased upon cold deacclimation at thermoneutrality (Figure 1B). As anticipated, the decrease in food intake upon reacclimating to thermoneutrality coincided with a rapid ∼55% decline in metabolic rate (VO_2_, Figure 1C). Metabolic rates remained slightly elevated during the first 2 days of deacclimating at thermoneutrality and gradually declined by an additional ∼15% by day 4 of deacclimation (Figure 1C). Respiratory exchange ratio (RER, Figure 1D) gradually increased during the deacclimation period, indicating a relative shift from lipid to carbohydrate substrate oxidation. Furthermore, the relative shift from an RER of ∼0.93 during the cold acclimation period to an RER >1 during cold deacclimation at thermoneutrality indicates that lipogenesis has been initiated. While total activity of mice in the chambers was not different in response to the acclimation protocol, ambulatory activity was low throughout cold exposure and increased during deacclimation. (Figures 1E and 1F).^25^

### Mitochondrial oxidative capacity and content regress in parallel during cold deacclimation

**Figure 2:**
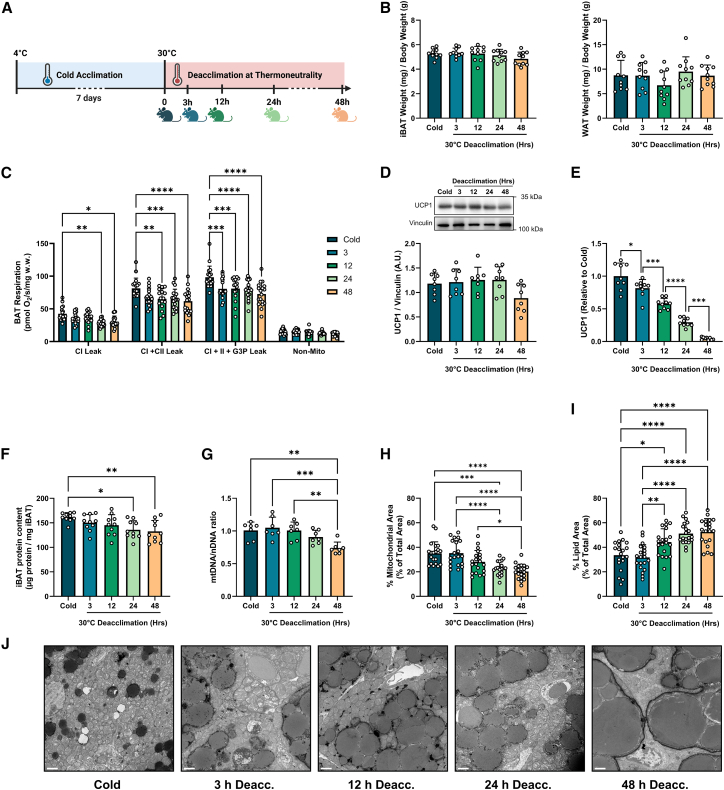
Capacity for mitochondrial uncoupling and mitochondrial content regress in parallel during cold deacclimation (A) Schematic summary of the time points for sample collection. C57BL/6J mice were acclimated to the cold (4°C) for 7 days with *ad libitum* access to food and water and subsequently transferred to thermoneutrality (30°C) for 3 h, 12 h, 24 h, or 48 h. Diagram created with BioRender.com. (B) High-resolution respiratory flux per mg of saponin-permeabilized iBAT. Uncoupled respiration was measured in the presence of oligomycin and octanoylcarnitine. Complex I (CI) leak respiration was measured after the addition of malate-pyruvate-glutamate, and CI + CII leak and CI + II + G3P leak respiration were measured following the sequential additions of succinate and glycerol-3-phosphate for respectively, (*n* = 16–18/group). (C) Immunoblotting of UCP1 protein expression (*n* = 8/group). (D) Quantitative PCR (qPCR) of *Ucp1* gene expression, (*n* = 9/group). (E) BAT protein concentration (per mg tissue) was measured using a BCA assay in protein lysates, (*n* = 10/group). (F) qPCR was used to determine the mtDNA:nDNA ratio (mt-ND1:n-HK2) (*n* = 7/group). (G–J) TEM images were analyzed by quantitative morphometry for (G) mitochondrial surface area and (H) lipid droplet surface area, (J) Representative TEM images, (*n* = 5 TEM images from 4 mice/group). Scale bars represent 2 μm. See also Figure S1. Comparisons between time points were determined using a one-way ANOVA with Tukey post-hoc tests, ∗*p* < 0.05, ∗∗*p* < 0.01, ∗∗∗*p* < 0.001, ∗∗∗∗*p* < 0.0001. All values are presented as means ± SD.

To elucidate mitochondrial functional and structural aspects involved in BAT metabolic remodeling, we acclimated C57BL/6J mice to the cold (4°C) with *ad libitum* access to food and water for 7 days and subsequently transferred them to thermoneutrality (30°C) for 3 h, 12 h, 24 h, or 48 h (Figure 2A). We selected these time points, as metabolic remodeling is initiated within the first 3 days of temperature acclimation.^25^ Deacclimation at thermoneutrality did not impact body weights or the weights of interscapular BAT (iBAT) or inguinal white adipose tissue (Figures 2B and S1A–S1C).

We sought to determine the functional changes in iBAT oxidative capacity using high-resolution respirometry (HRR) conducted on saponin-permeabilized iBAT. To assess respiration attributable to mitochondrial proton leak uncoupling, HRR assays were conducted in the presence of oligomycin and octanoyl carnitine to support fatty acid-driven uncoupled respiration. Upon deacclimating from the cold at thermoneutrality, decreases in CI + CII leak respiration were apparent by 12 h of deacclimation, and complex I (CI) leak respiration gradually declined by 24 h (Figure 2C). However, more rapid declines were observed in the leak respiration linked to the oxidation of glycerol-3-phosphate (G3P) by the FAD-linked mitochondrial glycerol-3-phosphate dehydrogenase (mGPDH), which were significant by 3 h (Figure 2C). Importantly, the activity of mGPDH is relatively high in iBAT compared to other tissues, and mGPDH is transcriptionally controlled by triiodothyronine (T3).^26^^,^^27^ As the esterification/acylation of G3P is a major rate-controlling step for the synthesis of glycerophospholipids and triacylglycerols,^28^ the rapid decline in G3P-supported respiration may indicate a shift toward lipid synthesis rather than oxidation.

Despite the decreases in leak respiration, protein expression of UCP1 did not differ between time points of cold deacclimation (Figure 2D). In contrast, *Ucp1* mRNA expression was rapidly downregulated (Figure 2E; primer pairs for targeted genes shown in Table 1). UCP1 protein has a half-life of ∼7 days *in vivo*, with differences paralleling changes in iBAT protein content.^29^ Indeed, decreases in iBAT protein content were evident after 48 h of cold deacclimation (Figure 2F). In line with the rapid downregulation in mitochondrial respiration, markers of BAT activation, *Dio2* and ELOVL fatty acid elongase 3 (*Elvol3*) decreased by 3 h of cold deacclimation, whereas regulators of BAT activity including PR domain containing 16 (*Prdm16*) and cell death inducing DFFA like effector a (*Cidea*) did not change across these selected time points of cold deacclimation (Figure S1D).

**Table 1:** Primer sequences used for quantitative PCR

Gene name	Forward sequence (5′–3′)	Reverse sequence (5′–3′)
Acly	AGGAAGTGCCACCTCCAACAGT	CGCTCATCACAGATGCTGGTCA
Acss2	GCTTCTTTCCCATTCTTCGGT	CCCGGACTCATTCAGGATTG
Acy1	TGCAACCCAATCCAGACTATG	GGCACCACCTCTATTTTCTGA
Acy3	ACACTGGAGTCTGCCTCATCTC	AGATTCCACGCTGAAGGTCTCC
Aspa	CCATATGAAGTGAGAAGGGCTC	CCTCAAGAATAAGAGTGCAACC
B2M	ATGCTGAAGAACGGGAAAAA	CGGCCATACTGGCATGCTTA
Cidea	TGCTCTTCTGTATCGCCCAGT	GCCGTGTTAAGGAATCTGCTG
Dio2	CAGTGTGGTGCACGTCTCCAATC	TGAACCAAAGTTGACCACCAG
Elovl3	TCCGCGTTCTCATGTAGGTCT	GGACCTGATGCAACCCTATGA
GAPDH	TCCCATTCTCGGCCTTGAC	ATGACTCCACTCACGGCAAATT
Hprt1	GCTGACCTGCTGGATTACAT	TTGGGGCTGTACTGCTTAAC
Nags	GCCTGCGGAATAACAGTCAGAAG	TCCACGATGAGCCGAATCTGCT
Nat8l	TGTGCATCCGCGAGTTCCGC	GCGGAAAGCCGTGTTGGGGA
Nrf1	CAACAGGGAAGAAACGGAAA	GCACCACATTCTCCAAAGGT
Nrf2	AGGTTGCCCACATTCCCAAACAAG	TTGCTCCATGTCCTGCTCTATGCT
Pgc1a	GTAGGCCCAGGTACGACAGC	GCTCTTTGCGGTATTCATCCC
Prdm16	CAGCACGGTGAAGCCATTC	GCGTGCATCCGCTTGTG
Ucp1	ACTGCCACACCTCCAGTCATT	CTTTGCCTCACTCAGGATTGG

Changes in iBAT mitochondrial content paralleled the declines in protein content, as indicated by decreases in the mtDNA/nDNA ratio, citrate synthase activity, and mitochondrial surface area and number in electron micrographs by 48 h of cold deacclimation (Figures 2G–2J, S1E, and S1G). The decreased mitochondrial content was likely due in large part to the rapid downregulation of PGC1α-mediated mitochondrial biogenesis, as evidenced by decreased *Pparg1ca* gene expression (Figure S1F). While gene expression of the nuclear respiratory factor 1 (*Nrf1*) did not differ in response to cold deacclimation, *Nrf2* was lower by 48 h of cold deacclimation (Figure S1F). As mitochondrial content decreased during cold deacclimation, lipid droplet size gradually expanded (Figures 2I, 2J, S1H, and S1I) likely owing to reduced lipolysis and triglyceride turnover.^30^

### Cold deacclimation induces extensive remodeling of BAT metabolism, including decreases in glutathione and TCA cycle metabolism

**Figure 3:**
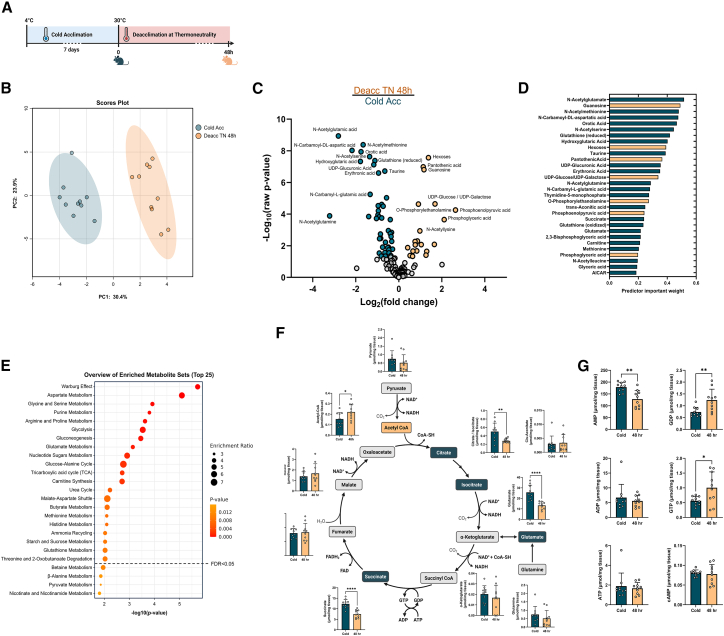
Metabolic profiling reveals cold deacclimation induces changes in the metabolism of amino acids, glutathione, and in TCA cycle activity (A) Schematic summary of the time points for sample collection for metabolomic analyses of BAT from cold acclimated and 48 h deacclimated mice, (*n* = 10/group). Diagram created with BioRender.com. (B) Principal component analysis of independent samples demonstrating separation between the BAT metabolome profiles. (C) Volcano plots of BAT metabolite profiles from cold acclimated mice and mice deacclimated at thermoneutrality for 48 h. Indicated in color are metabolites with *p* < 0.05 and log_2_ fold change over 1. Labeled are the top 20 metabolites. (D) Selection of the most significantly different features using the machine learning method ReliefF. (E) Metabolite set enrichment analyses of the metabolites altered between BAT from cold acclimated and deacclimated mice. (F and G) Quantitative analysis of individual metabolites relating to (F) the TCA cycle and (G) purine metabolism. Values in (G) are presented as means ± SD. Comparisons between groups were determined using Metaboanalyst. Comparisons for individual metabolites were determined using a two-tailed Student’s *t* test. ∗*p* < 0.05, ∗∗*p* < 0.01, ∗∗∗*p* < 0.001, and ∗∗∗∗*p* < 0.0001.

Studies assessing the iBAT metabolome during cold exposure and acclimation have demonstrated the profound capacity for the metabolic remodeling of iBAT. For example, there are rapid increases in the abundance and oxidation of fatty acids, amino acids, and glucose. To determine the impact of cold deacclimation, we assessed changes in the iBAT metabolome using quantitative ion-pairing liquid chromatography-mass spectroscopy (LCMS) in cold-acclimated mice and mice deacclimated at thermoneutrality for 48 h (Figure 3A). We chose to analyze BAT after 48 h of deacclimation to avoid measuring only acute stress responses (e.g., stress hormones, catecholamine changes) that occur during the initial deacclimation period and to capture dynamic changes in fuel utilization that paralleled the observed functional declines in mitochondrial leak respiration.

Principal-component analysis demonstrated clear separation of the clustered iBAT metabolite profiles from cold-acclimated mice and 48 h deacclimated mice (Figure 3B). After false discovery rate correction, we identified 53 out of 136 detected metabolites that differed between cold-acclimated and deacclimated mice (FDR<0.05) (Figure 3C). The machine learning feature selection method, ReliefF, identified N-acetyl amino acids (Glu, Met, Ser, and Gln), guanosine, orotic acid, and N-carbamoyl-DL-aspartic acid as key metabolites responsible for discriminating the cold-acclimated vs. cold-deacclimated BAT metabolic phenotypes (Figure 3D). Metabolite set enrichment analysis (MSEA) demonstrated that cold deacclimation induced changes in pathways related to amino acid metabolism (aspartate, glycine/serine), glycolysis/gluconeogenesis, purine and nucleotide sugar metabolism, and the TCA cycle (Figure 3E).

Substrate metabolism during cold deacclimation appears to favor glycogenesis and gluconeogenesis during cold-deacclimation, as evident by higher abundances of UDP-glucose and hexoses available for glycolysis entry and a lower ratio of pyruvate/lactate (Figures S2A and S2B). Similarly, analyses of TCA metabolites revealed decreased concentrations of citrate and succinate during cold deacclimation, suggesting an overall decrease in TCA cycle activity during the deacclimation period (Figure 3F). The increase in acetyl-CoA abundance in cold-deacclimated BAT further reflects a decrease in TCA cycle demand (Figure 3F). Lower succinate in cold-deacclimated BAT is interesting, given that accumulation of succinate can induce UCP1-mediated respiration in brown adipocytes, independent of adrenergic signaling.^20^ Moreover, purine nucleotides ATP, ADP, GTP, and GDP can bind directly to UCP1 and inhibit proton leak uncoupling. Consistent with decreased UCP1 activity, the abundance of both GDP and GTP was higher in BAT from cold-deacclimated mice, whereas AMP decreased in abundance in cold deacclimated BAT (Figure 3G).

Other notable differences include increased abundance of pantothenic acid in cold-deacclimated BAT (Figure S2C), indicating increased availability of CoA. Pantothenic acid is required for adipocyte browning and the induction of UCP1 expression.^31^^,^^32^ Pantothenate kinase 1 (PANK1), the rate-limiting enzyme for CoA synthesis, correlates with UCP1 expression in human BAT.^33^^,^^34^ Glutathione and metabolites such as serine and methionine, which are involved in the synthesis of cysteine, the rate-limiting amino acid in glutathione synthesis, were lower in cold-deacclimated BAT (Figure S2D). ROS levels are high in BAT during UCP1-mediated mitochondrial uncoupling,^35^ and thus, the decreased abundance of GSH-related metabolites likely reflects the decreased need for the antioxidant properties of GSH during cold deacclimation. The abundance of carnitine decreased during cold deacclimation (Figure S2E), which, together with the decrease in citrate, potentially indicates a decrease in futile fatty acid cycling. Indeed, gene expression of the *de novo* lipogenesis enzyme ATP-citrate lyase (ACLY), which generates acetyl-CoA from citrate, decreased by 48 h of cold deacclimation (Figure S2F).^36^^,^^37^ However, gene expression of acetyl-CoA synthetase 2 (ACSS2), which produces acetyl-CoA from acetate for lipid synthesis, did not change during the cold deacclimation period (Figure S2G). Notably, this futile cycling of fatty acid synthesis and fatty acid oxidation (FAS-FAO) is unlikely to change the absolute levels of certain metabolites, such as acetyl-CoA, but instead affects the metabolic flux (i.e., how they are synthesized and catabolized).^38^

### Aminoacylase 1 may regulate the abundance of N-acetylated amino acids during cold acclimation and deacclimation

**Figure 4:**
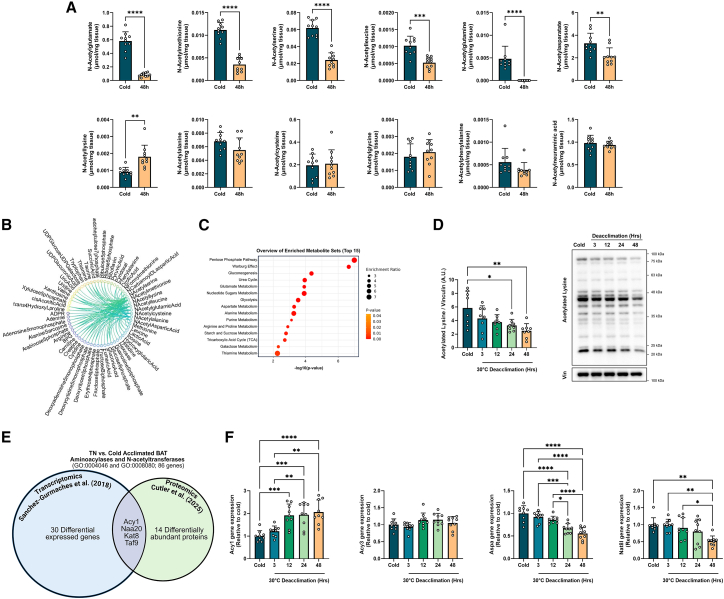
Aminoacylase 1 likely regulates the abundance of N-acetylated amino acids during cold acclimation and deacclimation (A) Individual metabolite abundance of select N-acetylated amino acids. Comparisons were determined using a two-tailed Student’s *t* test. (B) Distance correlations of the N-acetylated amino acids relating to the other measured metabolites, (*n* = 10/group). Shown are metabolites that have a significant difference between iBAT from cold and 48 h deacclimated mice, where in 48 h deacclimated BAT they have correlation over 0.7 (*p* < 0.003) with any of the N-acetylated-AAs and no such correlation in the cold BAT. (C) Metabolite set enrichment analyses of the metabolites that correlate with the N-acetylated amino acids at 48 h of cold deacclimation, but not in cold exposed BAT. (D) Immunoblotting of acetylated lysine residues on proteins, (*n* = 8/group). Comparisons between time points were determined using a one-way ANOVA with Tukey post-hoc tests. (E) Bioinformatic analysis of published RNA-seq^39^ and proteomics^40^ datasets to identify proteins involved in the (de)acetylation of proteins and amino acids during cold acclimation using the GO terms N-acetyltransferase activity (GO:0008080) and aminoacylase activity (GO:0004046). Four proteins were shared between datasets: aminoacylase 1 (Acy1), Nα-acetyltransferase 20 (Naa20), lysine acetyltransferase 8 (Kat8), and TATA-box binding protein associated factor 9 (Taf9). (F) Quantitative PCR (qPCR) of aminoacylase 1 (Acy1) and 3 (Acy3), aspartoacylase (Aspa), and N-acetyltransferase 8-like (Nat8l), (*n* = 9/group). Comparisons between time points were determined using a one-way ANOVA with Tukey post-hoc tests. All values are presented as means ± SD. ∗*p* < 0.05, ∗∗*p* < 0.01, ∗∗∗*p* < 0.001, ∗∗∗∗*p* < 0.0001.

N-acetyl amino acids were the top metabolites altered during cold-deacclimation (Figure 4A). Tracing experiments using [13C]-glucose in iBAT of mice acclimated at cold, room, or thermoneutral temperatures have similarly identified N-acetylated amino acids as thermogenic reporter metabolite candidates, and the observed [13C]-glucose labeling at M+2 suggests that the acetyl group is derived from glucose.^21^ Distance correlation analysis between N-acetyl amino acids and all other metabolites revealed that the abundances of N-acetyl amino acids at 48 h of deacclimation strongly correlated with metabolites involved in the pentose phosphate pathway and gluconeogenesis (Figures 4B and 4C).

High levels of N-acetylated amino acids during cold exposure may arise from the proteolytic degradation of N-terminal acetylated proteins or the transfer of an acetyl group to free amino acids via N-acetyltransferases. Thus, we sought to explore the mechanisms underlying the rapid change in abundance of N-acetyl amino acids. Interestingly, N-acetyllysine was the only N-acetylated amino acid to increase in abundance with cold-deacclimation (Figure 4A). This increase in N-acetyllysine corresponded with a decrease in protein lysine acetylation during cold deacclimation (Figure 4D), indicating an increase in protein degradation throughout the cold deacclimation period. Thus, we hypothesized that the decrease in N-acetylated amino acids during cold deacclimation was due to decreased activity of N-acetyltransferases, possibly combined with increased activity of zinc-dependent aminoacylases (ACY), which can deacetylate a wide range of Nα-acetylated amino acids, by hydrolyzing N-acetyl L-amino acids into free amino acids.^41^

We then leveraged publicly available published RNA-seq^39^ and proteomics^40^ datasets to identify proteins involved in the (de)acetylation of proteins and amino acids during cold acclimation using the GO terms N-acetyltransferase activity (GO:0008080) and aminoacylase activity (GO:0004046). Of the 86 genes mapped to these terms, 30 genes were differentially expressed, and 14 proteins differed in abundance (Figure 4E; Table S1). Four proteins were shared between datasets: aminoacylase 1 (Acy1) was lower in BAT from cold acclimated vs. TN mice, whereas the catalytic subunit of NatB complex, Nα-acetyltransferase 20 (Naa20), lysine acetyltransferase 8 (Kat8), and TATA-box binding protein associated factor 9 (Taf9) were higher in BAT from cold acclimated vs. TN (Figure 4E; Table S1).

We concentrated on aminoacylase expression, as ACY1 is strongly associated with the abundances of several N-acetyl amino acids in human plasma.^42^^,^^43^ There are 3 family members, which differ in amino acid specificity: ACY1 deacetylates neutral aliphatic N-acyl and N-acetyl-α-amino acids and mercapturic acids; ACY2 or aspartoacylase (ASPA) specifically deacetylates N-acetyl-aspartate; and ACY3 preferentially deacetylates Nα-acetylaromatic amino acids. Gene expression of *Acy1* increased during cold deacclimation, whereas *Aspa* decreased during cold deacclimation (Figure 4F), whereas *Acy3* expression did not change during cold deacclimation. Gene expression of *Aspa* decreased during cold deacclimation, which corresponded with reduced expression of N-acetyltransferase 8-Like (*Nat8l*), which synthesizes N-acetylaspartate from acetyl-CoA and aspartate (Figure 4F). Taken together, these data indicate that aminoacylase expression may be sensitive to environmental temperature and may contribute to the marked decreases in N-acetylated metabolites.

## Discussion

BAT is characterized by significant morphological and functional plasticity in response to changes in ambient temperatures. Most studies have focused on processes occurring during cold acclimation, and relatively little is known about mechanisms elicited during deacclimation from the cold. Here, we conduct a time-resolved analysis of iBAT deactivation during the transition to thermoneutrality following 7 days of cold acclimation. Our results demonstrate that whole-body metabolism rapidly decreases when cold-acclimated mice are deacclimated at thermoneutrality, and analyses of iBAT structure and function revealed the gradual remodeling of BAT mitochondria. Analyses of the iBAT metabolome highlighted the marked alterations in amino acid metabolism, glutathione, and TCA cycle between cold acclimated and 48 h deacclimated mice. Striking decreases in N-acetylated amino acids in 48 h deacclimated BAT indicate that select N-acetylated amino acids may act as thermogenic biomarkers and are possibly regulated by temperature-induced alterations in aminoacylase expression.

The duration of the cold exposure affects the pace at which BAT is inactivated when animals return to warm environments, with longer periods of cold acclimation often resulting in slower regression of BAT, with full deacclimation occurring after 2–3 weeks.^44^^,^^45^ The absence of sympathetic input is a homeostatic mechanism that halts thermogenic activation. Upon returning to a warm environment after a period of cold acclimation, decreased sympathetic signaling leads to reduced blood flow and induces dynamic metabolic remodeling of BAT.^46^^,^^47^ However, cold-induced metabolic adaptations may persist for several days after the cold exposure period has concluded, such as transient improvements in glucose homeostasis and redox homeostasis.^48^^,^^49^^,^^50^^,^^51^^,^^52^^,^^53^ Despite immediate drops in whole-body energy expenditure, our data demonstrate that higher tissue metabolic activity persists for 2 days when mice are deacclimated at thermoneutrality, further highlighting the metabolic costs of adaptations from cold acclimation. Studies in humans have similarly observed that many of the cold-induced metabolic effects do not immediately subside fully during the initial re-warming period.^54^ Specifically, sustained increases in glucose uptake, heat production, and substrate oxidation remain elevated for several hours to days after returning to warm environments.^54^^,^^55^ The observed hyperphagic response in cold-acclimated mice is thought to be a result of substantially increased metabolic demand to maintain energy reserves for oxidation and to induce a degree of diet-induced thermogenesis via the thermic effect of feeding, which provide additional thermogenic effects.^56^^,^^57^^,^^58^^,^^59^ Our finding that food intake decreases within the first 24 h of cold deacclimation in a thermoneutral environment is consistent with the important role of neuronal control of food intake to maintain energy homeostasis. Similarly, lower ambulatory and rearing activity in the cold may be attributable to behavioral and physiological responses to maintain body temperature by limiting or expanding body surface area exposed to ambient temperatures.^60^ In contrast to the immediate declines in whole-body energy expenditure, permeabilized iBAT did not exhibit immediate decreases in capacity for uncoupled mitochondrial respiration. Blood flow and glucose uptake in iBAT rapidly decrease in BAT within 2–6 h,^46^^,^^47^ suggesting that the decreased supply of oxygen and substrates to BAT mitochondria may limit *in vivo* BAT metabolic capacity, beyond any intrinsic declines in mitochondrial capacity. Indeed, BAT oxygen consumption strongly correlates with blood flow to BAT.^33^^,^^61^^,^^62^ The functional changes in mitochondrial oxidative capacity following the cessation of cold-induced thermogenesis appear to coincide with the gradual declines in mitochondrial content, UCP1 protein expression and BAT protein content, which is consistent with previous findings.^29^^,^^44^^,^^53^^,^^63^^,^^64^ The decrease in mitochondrial content likely contributes to the corresponding declines in uncoupled mitochondrial respiration. The declining BAT mitochondrial content observed during cold deacclimation is accompanied by increases in unilocular lipid droplet formation and glycogen repletion,^44^^,^^52^^,^^65^^,^^66^ consistent with decreased demand for substrate oxidation. Together, these results suggest that a decreased supply of substrates for oxidation, for example, due to decreased supply from the circulation or to decreased sympathetic nervous system stimulation of cellular lipolysis, may drive immediate temperature-induced decreases in BAT oxidative capacity and metabolic remodeling. Moreover, the ability of thermogenesis to be rapidly switched on and off in response to changes in environmental temperature is independent of the mechanisms responsible for the gradual remodeling of BAT during cold (de)acclimation (e.g., protein degradation, mitochondrial turnover).

Sophisticated *in vivo* metabolite tracing experiments have demonstrated that glucose and fatty acids primarily fuel BAT during thermogenesis.^67^^,^^68^^,^^69^ Cytosolic fatty acid synthesis also increases during thermogenesis, resulting in the futile cycling of fatty acids.^70^ Citrate generated by the TCA cycle is exported to the cytosol via the citrate-malate shuttle and subsequently metabolized by ATP-citrate lyase (ACLY) to synthesize acetyl-CoA for *de novo* lipogenesis via acetyl-CoA carboxylase (ACC1) and fatty acid synthase (FASN).^37^^,^^66^^,^^71^ The resulting acyl-carnitines can then return to the mitochondria for oxidation.^37^ Our observations of decreased citrate and carnitine abundance and decreased gene expression of ATP-citrate lyase by 48 h of deacclimation are in line with the notion that the futile cycling between fatty acid synthesis and oxidation is reduced following the cessation of the cold stimulus. The rapid changes in BAT metabolic capacity precede the molecular remodeling of brown adipocytes during cold-acclimation, consistent with the conclusion that metabolites could primarily drive the acute-phase response in BAT. Reporter metabolites are thought to represent key regulatory areas within a metabolic network that maintain energy homeostasis in response to environmental stressors.^72^ Alterations in the abundance of N-acetylated amino acids have been identified in cold-exposed BAT.^20^^,^^21^^,^^73^ Our finding that N-acetylated amino acids rapidly decrease in abundance during cold deacclimation further supports their role as potential metabolite biomarkers of thermogenesis.

While fatty acids are a major source of acetyl-CoA during cold exposure, M^+2^ labeling observed in [13-C]-glucose tracing experiments in cold-exposed mice suggests that the acetyl group of the N-acetyl-amino acids is mainly derived from glucose via glycolysis and pyruvate dehydrogenase activity.^21^ N-acyl amino acids, which have a fatty acid acyl moiety rather than an acetyl group have been shown to activate UCP1 and uncouple mitochondrial respiration in a similar manner as conventional fatty acids.^74^^,^^75^ In contrast, while N-acetylated amino acids have been identified in cold-exposed BAT,^20^^,^^21^^,^^73^ their role in thermogenesis is relatively unexplored.

High levels of N-acetylated amino acids during cold exposure are hypothesized to result from the proteolytic degradation of N-terminal acetylated proteins.^21^ Our findings that protein lysine acetylation and protein content decrease in BAT during cold deacclimation are consistent with the conclusion that cold deacclimation increases proteolytic degradation of proteins, giving rise to the observed increase in N-acetyllysine abundance. Notably, our data demonstrating increased aminoacylase 1 expression and decreased Nat8l expression suggest that N-acetyltransferase activity is high during cold exposure and decreases upon cold deacclimation at thermoneutrality, indicating lower amino acid catabolic flux to acetylated derivatives. Skeletal muscle uses a similar L-carnitine/carnitine acetyltransferase (CrAT) buffering system for mitochondrial acetyl-CoA, particularly during times of high nutrient availability.^76^ Thus, our data are consistent with the idea that N-acetyl amino acids may act as transport molecules to buffer high acetyl-CoA levels that can be converted to acetate for lipid synthesis^37^ during cold acclimation. Moreover, as demonstrated for N-acetyl aspartate in brown adipocytes, the catabolism N-acetyl amino acids may provide cytosolic acetyl-CoA, which may regulate histone acetylation during thermogenesis.^77^ Alternatively, N-acetyl amino acids may act as signaling molecules for organ cross-talk, as high levels of N-acetylglutamate and N-acetylaspartate have been observed in extracellular fluid derived from BAT.^73^

Individual N-acetylated amino acids may play specific roles in BAT during thermogenesis. For example, N-acetyl aspartate promotes lipid turnover, and can induce UCP1 expression in a PPARα-dependent manner.^78^ N-acetyl glutamate is a precursor for arginine biosynthesis and acts as an allosteric activator of the rate-limiting urea cycle enzyme, carbamoyl phosphate synthetase 1 (CPS1).^79^ Mechanistic studies have demonstrated that N-acetyl glutamate and N-acetyl methionine can impair mitochondrial membrane potential (ΔΨm) and induce mitochondrial swelling in calcium (Ca^2+^)-loaded mitochondria in a dose-dependent manner, indicating they may play a role in inducing the mitochondrial permeability transition pore (MPTP) opening.^80^ N-acetyl glutamate can inhibit glutamate oxidation by glutamate dehydrogenase (GDH) as well as isocitrate dehydrogenase activity (IDH).^81^ However, the specific roles of N-acetyl amino acids in BAT and beige adipose tissue during thermogenesis and deacclimation warrant further exploration.

In conclusion, our findings outline progressive changes in BAT structure and function occurring in the transition from a cold acclimated state to a thermoneutral environment. Specifically, high metabolic rates and food intake during the cold acclimated state rapidly decrease in a thermoneutral environment. More gradual were the progressive declines in BAT mitochondrial uncoupling capacity, protein content, and UCP1 expression, as well as decreases in mitochondrial content, confirmed by mtDNA/nDNA ratios and electron microscopy. Metabolomic profiling showed decreased N-acetylated amino acids and major alterations in pathways involving amino acid metabolism, purine nucleotides, and the TCA cycle. Enhanced gene expression of ACY1 and ACY3 suggest that enhanced aminoacylase activity may contribute to the marked decrease in N-acetylated metabolites during cold deacclimation. Together, these findings highlight the biochemical and metabolic processes involved in BAT thermogenesis and deactivation.

### Limitations of the study

Since the mice were originally housed at room temperature (22°C), direct comparisons cannot be appropriately made to the initial phenotype. Similarly, molecular analyses of BAT were studied up to 48 h of cold deacclimation, as such restitution of BAT past the selected time points has not been elucidated. Finally, mechanistic aspects and the functional significance of N-acetylated amino acid levels warrant further investigation.

## Resource availability

### Lead contact

Further information and requests for resources should be directed toward the lead contact, Mary-Ellen Harper (mharper@uottawa.ca).

### Materials availability

This study did not generate new, unique reagents.

### Data and code availability

All data generated for this manuscript are presented in the main manuscript or additional supporting files. Original western blot data are available in the Source Data file. This paper does not report the original code. Any additional information required to reanalyze the data reported in this paper is available from the lead contact upon request.

## Acknowledgments

The authors would like to thank Jian Ying Xuan for her excellent technical support, the Metabolomics Core, Cell Biology and Image Acquisition Core, Animal Care and Veterinary Services Behavioral Core, and the Histology Core, all funded by the University of Ottawa, Ottawa, Canada. The authors would also like to thank Kelly Sears, Hojatollah Vali, and Jeannie Mui at the Facility for Electron Microscopy Research of McGill University for help in microscope operation and data collection. This research was funded through a Discovery grant from the Natural Sciences and Engineering Research Council (10.13039/501100000038NSERC) of Canada.

## Author contributions

Conceptualization: C.A.P. and M.-E.H.; investigation: C.A.P., N.K., E.M., L.M.K.H., L.S.K., V.V., M.K., Z.E.H., and Y.B.; formal analysis: C.A.P., N.K., E.M., L.M.K.H., L.S.K., V.V., M.K., Z.E.H., and M.C.-C.; software: L.S.K. and M.C.-C.; supervision: M.-E.H.; funding acquisition: M.E.H.; writing – original draft: C.A.P.; writing – review & editing: C.A.P., N.K., E.M., L.M.K.H., L.S.K., V.V., M.K., Z.E.H., Y.B., M.C.-C., and M.-E.H.

## Declaration of interests

The authors declare no conflicts of interest.

## STAR★Methods

### Key resources table

**Table:** 

REAGENT or RESOURCE	SOURCE	IDENTIFIER
**Antibodies**		

Anti-Mouse IgG (H + L) Antibody, HRP Conjugated	Promega	Cat# W4021; RRID: AB_430834
Anti-Rabbit IgG (H + L), HRP Conjugate antibody	Promega	Cat# W4011; RRID: AB_430833
Anti-Acetylated Lysine Antibody	Millipore Sigma	Cat# ST1027; RRID:AB_10682447
Anti-UCP1 Antibody	Sigma Aldrich	Cat# U6382; RRID:AB_261838
Anti-Vinculin Antibody [EPR8185]	Abcam	Cat# ab129002; RRID:AB_11144129

**Chemicals, peptides, and recombinant proteins**		

Protease Inhibitor Cocktail	Sigma Aldrich	Cat# P8340
RIPA Lysis Buffer	Millipore	Cat# 20-188
SsoAdvanced™ Universal SYBR® Green Supermix	Bio-Rad	Cat# 1725270
SuperSignal™ West Pico PLUS Chemiluminescent Substrate	ThermoFisher	Cat# 34579

**Critical commercial assays**		

Pierce™ BCA Protein Assay Kits	ThermoFisher	Cat# 23225

**Software and algorithms**		

Biorender	Biorender	RRID: SCR_018361
GraphPad Prism 10.5	GraphPad Software	RRID: SCR_002798
ImageJ	National Institutes of Health	RRID: SCR_002285
Empanada-Napari	Conrad et al.^82^	https://empanada.readthedocs.io/en/latest/Napari; RRID: SCR_022765
MATLAB	MathWorks	RRID:SCR_001622
MetaboAnalyst	MetaboAnalyst	RRID:SCR_015539
R (v4.0.4)	R Core Team	RRID: SCR_001905

### Experimental model and study participant details

All experimental procedures were approved by the University of Ottawa Animal Care Committee (Protocol #4179) and conducted in accordance with the guidelines and principles of the Canadian Council of Animal Care. Mice were maintained on a C57BL/6J background (Jackson Laboratories) and housed in ventilated cages in a temperature-controlled room (22°C) on a 12/12 h light-dark cycle, with *ad libitum* access to a standard chow diet (44.2% carbohydrate, 6.2% fat, and 18.6% crude protein) and water. Prior to cold exposure, mice were individually housed for at least 3 days. Male and female mice (7-9 weeks old) were acclimated to the cold (4°C) for 7 days and subsequently deacclimated at 09:00 to thermoneutrality (30°C) where they remained for 0 hours (cold-acclimated controls), or for 3, 12, 24, or 48 hours of deacclimation. Unless otherwise stated, mice were sacrificed by cervical dislocation, and interscapular BAT (iBAT) was collected for subsequent analyses.

### Method details

#### Metabolic phenotyping

A 12-chamber comprehensive lab animal monitoring system (CLAMS; Columbus Instruments, Columbus, OH) was used to measure volitional activity, food intake, and whole-body energy metabolism (volume of O_2_ consumption (VO_2_), volume of CO_2_ production (VCO_2_), and respiratory exchange ratio (RER; VCO_2_/VO_2_)), with measurements recorded every 26 minutes. The CLAMS Oxymax system (Columbus Instruments) calculates VO_2_ and VCO_2_ using classic open-circuit indirect calorimetry gas equations applied to inlet/outlet gas fractions and measured airflow, with the unmeasured flow derived using the Haldane (nitrogen) transformation https://www.colinst.com/products/oxymax-clams.

Mice were acclimated to 4°C for 3 days in a temperature-controlled room prior to being transferred to individual CLAMS chambers maintained at 4°C with *ad libitum* access to food and water for 5 days. Data were collected only in the last 2 days of the cold period, after mice had become accustomed to the non-home cage environment. Subsequently, the ambient temperature in the CLAMS chambers was increased to 30°C for 4 days. As recorded by environmental CLAMS monitors, the transition from 4°C to 30°C took ∼45 minutes.

For activity measurements, ambulatory activity was calculated as the sum of ambulatory beam breaks in the x, y, and Z planes (XAMB, YAMB, ZAMB). Total activity was calculated as the sum of all beam breaks detected in the X, Y, and Z dimensions (XTOT, YTOT, ZTOT).

#### High resolution respirometry

Oxygen consumption was quantified in BAT using high-resolution respirometry (O2K, Oroboros Instruments, Innsbruck, Austria). Freshly isolated iBAT was minced and subsequently placed in ice-cold BIOPS buffer [BIOPS (in mM): 2.77 CaK_2_EGTA, 7.23 K_2_EGTA, 5.77 Na_2_ATP, 50 MES, 20 imidazole, 20 taurine, 15 PCr, 0.5 DTT, 6.56 MgCL_2_–6H_2_O, pH 7.1 at 0°C] and permeabilized with 50 μg/mL saponin for 30 min at 4°C under gentle agitation. Samples were then washed for 30 min in ice-cold mitochondrial respiration medium [MiR05 (in mM): 110 sucrose, 60 K-lactobionate, 20 HEPES, 20 taurine, 10 KH2PO4, 3 MgCl2, 0.5 EGTA, and 1 mg/ml BSA, pH 7.1 at 37°C]. Experiments were performed in duplicate at 37°C in 2 mL of mitochondrial respiration media. To evaluate uncoupled respiration, assays were performed in the presence of 5 μM oligomycin and 0.1 mM octanoylcarnitine to inhibit ATP synthase and activate uncoupled respiration. The assay protocol consisted of consecutive additions of 2 mM malate, 5 mM pyruvate, 10 mM glutamate (CI Leak), 10 mM succinate (CI+II Leak), 5 mM glycerol 3-phosphate (CI+II+cGpDH Leak) and Antimycin A (non-mitochondrial respiration).

#### Transmission electron microscopy (TEM) of BAT

Samples for TEM were processed and imaged by the Facility for Electron Microscopy Research at McGill University. Briefly, freshly dissected iBAT was fixed with 2.5% glutaraldehyde in 0.1 M sodium cacodylate buffer (pH 7.4). The tissue was washed for 3 x 10 minutes in 0.0.1 M sodium cacodylate buffer and post-fixed in 1% aqueous OsO4 +1.5% aqueous potassium ferrocyanide for 2 hours at 4°C. The samples were dehydrated using increasing ethanol concentrations, infiltrated with graded EPON:ethanol mixtures, and embedded in 100% Epon resin. Sections were prepared using Leica Microsystems UC6 Ultramicrotome and counterstained with uranyl acetate and Reynold’s lead. TEM grids were imaged by an FEI Tecnai G2 Spirit 120 kV cryo-TEM equipped with an AMT NanoSprint15 MK2 CMOS Camera.

Images from 5 micrographs/mouse were blinded for analysis. Mitochondria were semi-automatedly segmented and annotated using the Empanada-Napari plugin,^82^ by training the MitoNet deep learning model. Training patches were selected at random from the images using the built-in model MitoNet_v1 to run 2D inference, and the resulting segmentations were then manually corrected in Napari. MitoNet_v1 was trained on the proofread patches for 100 iterations to generate a fine-tuned model. Outcomes from the fine-tuned model were manually checked to correct errors to ensure accurate annotation. The number and surface area of mitochondria were measured using the napari-skimage-regionprops plugin. Analysis of lipid droplet size and number were blinded and manually performed in ImageJ.

#### Protein extraction and immunoblotting

Frozen iBAT was homogenized in ice-cold RIPA buffer (Millipore) supplemented with a protease inhibitor cocktail (Sigma) and Phosphatase inhibitor cocktail (Thermofisher) using a bead mill homogenizer (Fisherbrand). The homogenates were centrifuged at 10,000 g for 10 min at 4°C, and the resulting top solidified lipid layer was discarded and the infranatant layer was carefully collected and centrifuged again at 10,000 g for 10 min at 4°C to remove any remaining lipids. Protein concentration was determined using a commercially available bicinchoninic acid kit per the manufacturer's protocol (ThermoFisher). Proteins were separated by SDS-PAGE under reducing conditions and transferred to PVDF membranes and incubated overnight with primary antibodies against UCP1 (1:6000; Sigma Aldrich, #U6382) and acetylated lysine-containing proteins (1:2000, Millipore Sigma, #ST1027). Vinculin (1:5000, Abcam, ab129002) was used as a loading control. Protein bands were visualized using the ChemiDoc™ MP Imaging System (Bio-Rad) and densitometry band analyses were performed using ImageJ (NIH).

#### Citrate synthase activity

Citrate synthase activity was determined in iBAT protein lysates as previously described.^83^ The change in the rate of absorbance at 412 nm and pathlength was measured using a BioTek Synergy Mx Microplate Reader (BioTek Instruments) in the presence of 50 mM Tris-HCl (pH 8.0), with 0.2 mM DTNB, 0.1 mM acetyl-CoA and 0.25 mM oxaloacetate. Enzyme activity was calculated using the extinction coefficient of 13.6 mM^−1^cm^−1^ for citrate synthase.

#### Mitochondrial and nuclear DNA quantification

DNA was extracted from frozen iBAT as previously described.^84^ SsoAdvanced™ Universal SYBR® Green Supermix (Bio-Rad) was used for quantitative PCR on the CFX96 Real-Time PCR Detection System (Bio-Rad). Mitochondrial DNA (mtDNA) to nuclear DNA (nDNA) ratios were determined through qPCR against mitochondrial DNA gene mt-ND1 (FWD: 5′-CTAGCAGAAACAAACCGGGC-3′, REV: 5′-CCGGCTGCGTATTCTACGTT-3′) and nuclear DNA gene HK2 (FWD: 5′-GCCAGCCTCTCCTGATTTTAGTGT-3′, REV: 5′-GGGAACACAAAAGACCTCTTCTGG-3′).

#### RNA extraction and quantitative PCR

RNA was extracted from frozen iBAT using Trizol (ThermoFisher, #15596026) according to the manufacturer’s instructions. Total RNA concentration was measured using a NanoDrop™ 2000 UV–Vis spectrophotometer (Thermo Scientific). cDNA was synthesized using the All-In-One 5X RT MasterMix (ABM, #G592). Quantitative PCR was performed using the SsoAdvanced Universal SYBR Green Supermix (Bio-Rad, #1725272) and run on the CFX96 (Bio-Rad). Primer pairs for target genes are outlined in Table 1. As a control for between-sample variability, mRNA levels were normalized to the geometric mean of beta-2 microglobulin (B2M), hypoxanthine phosphoribosyltransferase 1 (Hrpt1), glyceraldehyde-3-phosphate dehydrogenase (GAPDH). Relative transcript expression was calculated using the 2^−ΔΔCt^ method.^85^

#### Targeted metabolomics

Mice were fasted for 2 hours prior to being anesthetized with isoflurane and placed on ice. Cardiac perfusion was then performed with ice-cold 150 mM ammonium formate (pH 7.4, 0.25 ml/min for 6 min) to remove blood from tissues. BAT was rapidly isolated, snap-frozen in liquid nitrogen, and stored at −80°C until metabolomics processing.

Frozen iBAT was homogenized using a bead mill homogenizer at 4°C (Fisherbrand Bead Mill 24 Homogenizer) in a −20°C equilibrated solution containing methanol, water, and acetonitrile (OmniSolv, Sigma). Homogenates were then incubated with a 2:1 dichloromethane:water solution on ice for 10 min. The polar and non-polar phases were separated by centrifugation at 3200 x g for 10 min at 1°C. The upper polar phase was evapourated using a refrigerated CentriVap Vacuum Concentrator at −4°C (LabConco Corporation, Kansas City, MO). Samples were run on an Agilent 6470A tandem quadrupole mass spectrometer equipped with a 1290 Infinity II ultra-high performance LC (Agilent Technologies) utilizing the Metabolomics Dynamic MRM Database and Method (Agilent), using ion-pairing reverse phase chromatography.^86^ This method was further optimized for phosphate-containing metabolites with the addition of 5 μM InfinityLab deactivator (Agilent Technologies) to mobile phases A and B, which requires decreasing the backflush acetonitrile to 90%. Multiple reaction monitoring (MRM) transitions were optimized using authentic standards and quality control samples. Metabolites were quantified by integrating the area under the curve of each compound using external standard calibration curves with Mass Hunter Quant (Agilent). No corrections for ion suppression or enhancement were performed; as such, uncorrected metabolite concentrations are presented. Further data processing analysis was conducted in MetaboAnalyst 6.0.^87^ Metabolite set enrichment analysis (MSEA) was performed using over-representation analysis with the Small Molecule Pathway Database as a library.

#### Data mining and network analysis

Data mining and network analyses were performed using in-house software written in Matlab 2024b (Matworks Inc) and https://complimet.ca/sidco.^88^ ReliefF was used for feature selection (command relief running under Matlab) using Euclidean distances as a metric for feature similarity and the 200 nearest neighbors for weight assessment. Correlation analysis using the distance correlation calculations were performed using SIDCO^88^ and in-house routines developed in Matlab.^89^^,^^90^ Correlation p-values were calculated using Student's t cumulative distribution function. Metabolite differences between groups were determined using linear regression comparisons of correlation values in the two groups for each metabolite, as previously described.^90^

#### Bioinformatic analyses of iBAT transcriptomics and proteomics

Publicly available published datasets from BAT cold exposure and cold acclimation studies were identified for analyses of aminoacylase and N-acetyltransferase expression. Analysed transcriptomic data from mice housed at thermoneutrality or cold (4°C) for 4 weeks were obtained from Sanchez-Gurmaches et al. (2018)^39^ supplementary files. Proteomics data were obtained from Cutler et al. (2025),^40^ where mice were housed under thermoneutral (30°C) or cold (5°C) conditions for 3 weeks. To identify proteins involved in the (de)acetylation of proteins and amino acids during cold acclimation, the GO terms (https://www.ebi.ac.uk/QuickGO/) N-acetyltransferase activity (GO:0008080) and aminoacylase activity (GO:0004046) were used to select targets with an FDR<0.05 as computed by the authors.

### Quantification and statistical analysis

Statistical analyses were performed using GraphPad Prism 10 (GraphPad Prism, La Jolla, CA, USA) and all values are reported as mean ± SD with a significance level of *p* < 0.05. For metabolic phenotyping analyses performed with CLAMS, a one-way repeated measures (RM) ANOVA was used to determine the effects cold-deacclimation. For subsequent experiments using cold acclimated (4°C) and cold deacclimated (30°C) mice at varying timepoints (3, 12, 24, or 48 hours of deacclimation), a one-way ANOVA was used to determine the effects cold-deacclimation. Tukey post-hoc tests and multiple comparisons tests were used to assess changes between the timepoints of cold-deacclimated state. For metabolomic comparisons between cold acclimated and 48 h cold deacclimated mice, a two-tailed Student’s t-test was used. Statistical details for experiments are provided in the figure legends.

## Supplemental information

Document S1. Figures S1 and S2

Data S1. Uncropped western blots, related to Figures 2D and 4D

Data S2. Metabolomics data, related to Figures 3 and 4

Table S1. Bioinformatic analysis of published RNA-seq and proteomics datasets, related to Figure 4E
