# Supplementary material for: Functional and metabolomic analyses of brown adipose tissue during cold-deacclimation reveal rapid N-acetylated amino acid adaptations
Source: iScience. 2026 Feb 25;29(4):115146. doi: 10.1016/j.isci.2026.115146 (PMC12999349; doi:10.1016/j.isci.2026.115146)
Supplement: Document S1. Figures S1 and S2 [file mmc1.pdf]

iScience, Volume *29*

Supplemental information

Functional and metabolomic analyses of brown adipose tissue during cold-deacclimation reveal rapid N-acetylated amino acid adaptations

Chantal A. Pileggi, Ella McIlroy, Lauren M.K. Hamilton, Nidhi Kuksal, Luke S. Kennedy, Valeria Vasilyeva, Michel N. Kanaan, Ziyad El Hankouri, Yan Burelle, Miroslava Cuperlovic-Culf, and Mary-Ellen Harper

Supplementary Information

Table S1: Published RNA-seq [40] and proteomics [41] datasets identifying proteins involved in the (de)acetylation of proteins and amino acids during cold acclimation using the GO terms N-acetyltransferase activity (GO:0008080) and aminoacylase activity (GO:0004046).

Figure S1

(A) Body weights of mice prior to the cold acclimation period (RT). Mice were then acclimated to the cold (4°C) for 7 days, and subsequently transferred to thermoneutrality (30°C) for 3 h, 12 h, 24 h, or 48 h, (n=10/group).

(B) Brown adipose tissue weight, (n=10/group).

(C) White adipose tissue (epididymal) weight, (n=10/group).

(D) qPCR was used to determine the relative transcript levels of BAT activation markers, PR domain containing 16 (*Prdm16*), cell death inducing DFFA like effector a (*Cidea*), type 2 iodothyronine deiodinase (*Dio2*), and ELOVL fatty acid elongase 3 (*Elvol3*), (n=9/group).

(E) Citrate synthase activity, (n=8-10/group).

(F) qPCR was used to determine the relative transcript levels of mitochondrial biogenesis transcription factors including PGC1α (Ppargc1a), nuclear factor 1 (NRF) and 2 (NRF2), (n=9/group).

(G-I) TEM images were analyzed by quantitative morphometry for **(G)** Number of mitochondria, **(H)** number of lipid droplets, and **(I)** average lipid droplet size, (n=5 TEM images from 4 mice/group). See also Figure 2.

Comparisons between timepoints were determined using a one-way ANOVA with Tukey post-hoc tests, *<$p<0.05$ **$p<0.01$, ***$p<0.001$, ****$p<0.0001$. All values are presented as means ± SD.

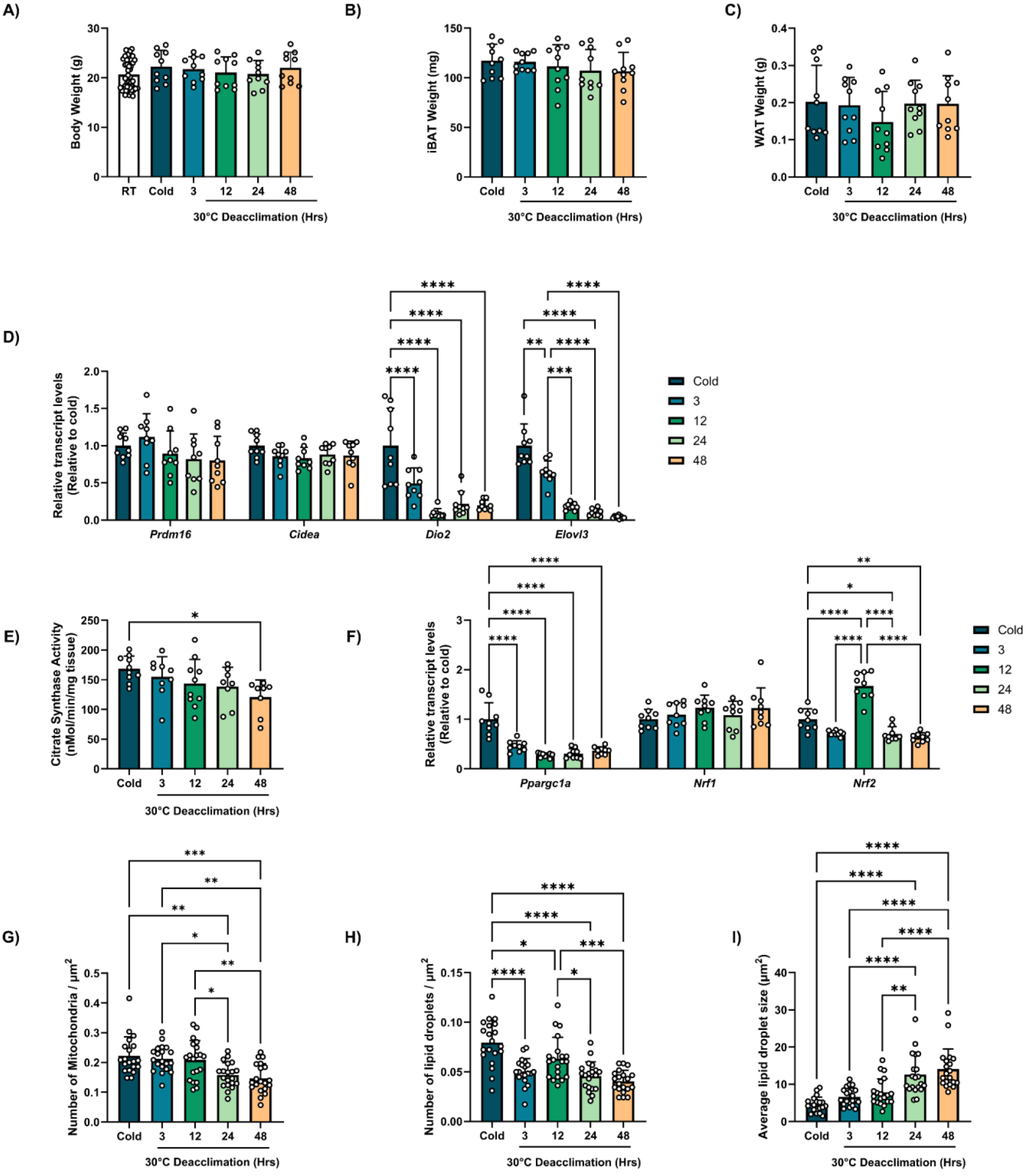

A)
Body Weight (g)
RT
Cold
3
12
24
48
30°C Deacclimation (Hrs)
B)
iBAT Weight (mg)
C)
WAT Weight (g)
D)
Relative transcript levels (Relative to cold)
Prdm16
Cidea
Dio2
Elovl3
Cold
3
12
24
48
E)
Citrate Synthase Activity (nMol/min/mg tissue)
F)
Ppargc1a
Nrf1
Nrf2
G)
Number of Mitochondria / µm²
H)
Number of lipid droplets / µm²
I)
Average lipid droplet size (µm²)

Figure S2

(A) Quantitative analysis of individual metabolites relating to glycolysis, (n=10/group).

(B) The ratio of pyruvate to lactate, (n=10/group).

(C) Quantitative abundance of pantothenate, (n=10/group).

(D) The abundances of serine, methionine, S-adenosyl homocysteine (SAH), glutamate, gamma-glu-cys, oxidized glutathione (GSSG) and reduced glutathione (GSH) (relative to cold), (n=10/group).

(E) Quantitative abundance of carnitine, (n=10/group).

(F-G) qPCR of gene expression of **(F)** ATP citrate lyase and **(G)** acetyl-CoA synthetase 2, (n=9/group). Comparisons between timepoints were determined using a one-way ANOVA with Tukey post-hoc tests.

Comparisons for individual metabolites were determined using a two-tailed Student's t-test. All values are presented as means ± SD. *<p<0.05 **p<0.01, ***p<0.001, ****p<0.0001.

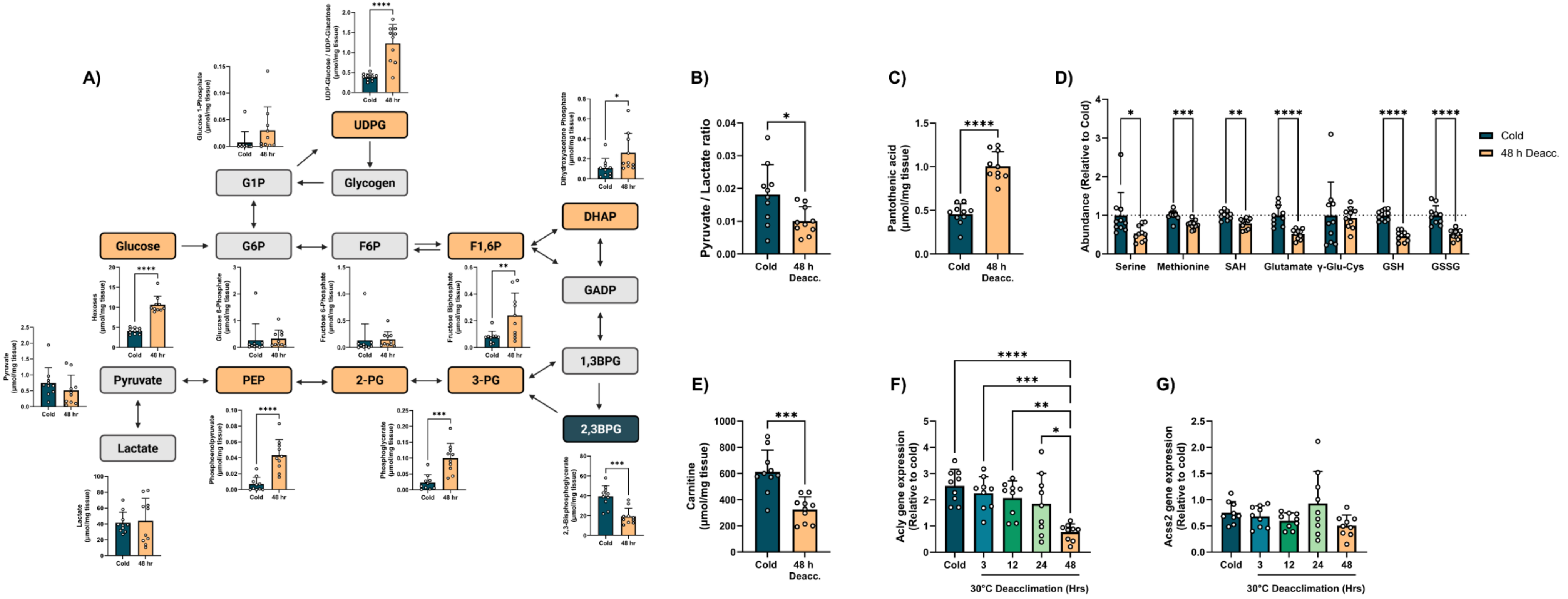
A)
UDPG
Glycogen
G1P
Glucose
G6P
F6P
F1,6P
DHAP
GADP
1,3BPG
2,3BPG
Pyruvate
PEP
2-PG
3-PG
Lactate
Cold
48 hr
UDP-Glucose / UDP-Glacatose (µmol/mg tissue)
Glucose 1-Phosphate (µmol/mg tissue)
Dihydroxyacetone Phosphate (µmol/mg tissue)
Hexoses (µmol/mg tissue)
Glucose 6-Phosphate (µmol/mg tissue)
Fructose 6-Phosphate (µmol/mg tissue)
Fructose Biphosphate (µmol/mg tissue)
Pyruvate (µmol/mg tissue)
Lactate (µmol/mg tissue)
Phosphoenolpyruvate (µmol/mg tissue)
Phosphoglycerate (µmol/mg tissue)
2,3-Bisphosphoglycerate (µmol/mg tissue)
B)
Pyruvate / Lactate ratio
Cold
48 h Deacc.
C)
Pantothenic acid (µmol/mg tissue)
D)
Abundance (Relative to Cold)
Serine
Methionine
SAH
Glutamate
γ-Glu-Cys
GSH
GSSG
Cold
48 h Deacc.
E)
Carnitine (µmol/mg tissue)
F)
Acly gene expression (Relative to cold)
Cold
3
12
24
48
30°C Deacclimation (Hrs)
G)
Acss2 gene expression (Relative to cold)
