# Supplementary material for: Functional and metabolomic analyses of brown adipose tissue during cold-deacclimation reveal rapid N-acetylated amino acid adaptations
Source: iScience. 2026 Feb 25;29(4):115146. doi: 10.1016/j.isci.2026.115146 (PMC12999349; doi:10.1016/j.isci.2026.115146)
Supplement: Data S1. Uncropped western blots, related to Figures 2D and 4D [file mmc2.pdf]

Figure/Target	
**Figure 2D**<br>UCP1	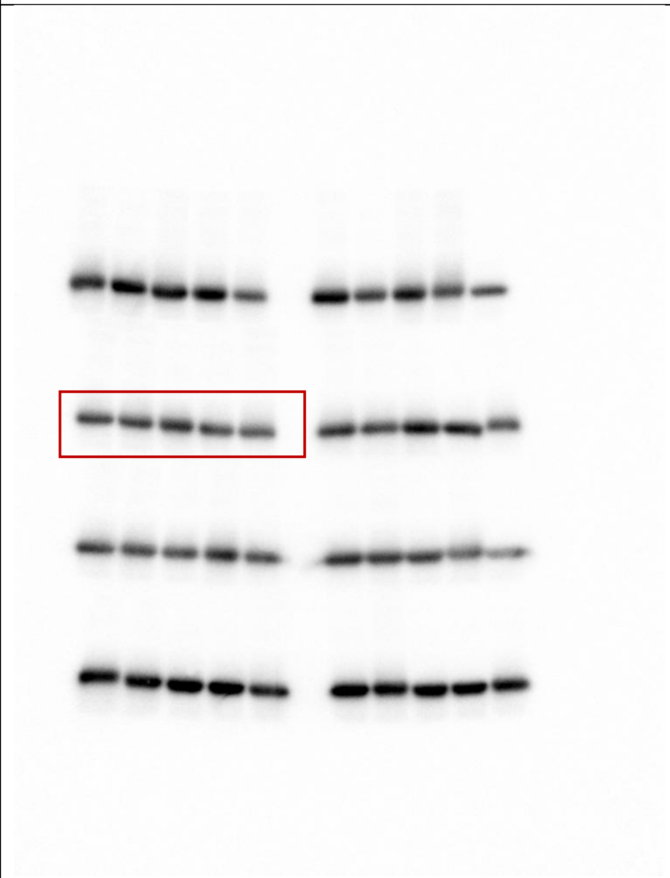
**Figure 2D**<br>Vinculin for UCP1	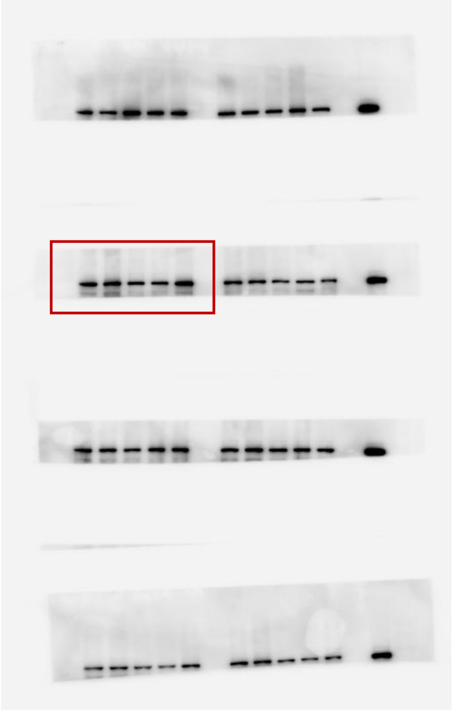

**Figure 4D**<br>Acetyl Lysine<br>(Gel 1 and 2)	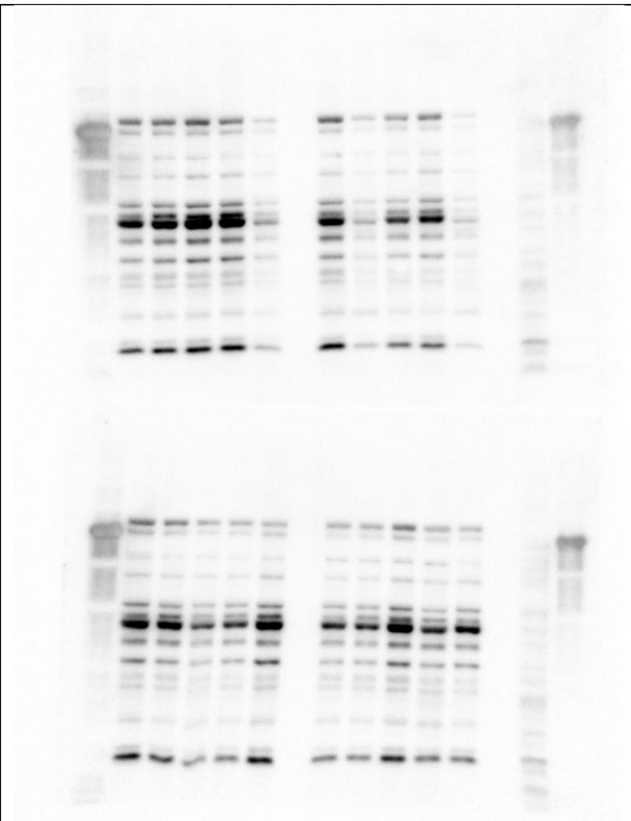
**Figure 4D**<br>Vinculin for Acetyl Lysine (Gel 1 and 2)	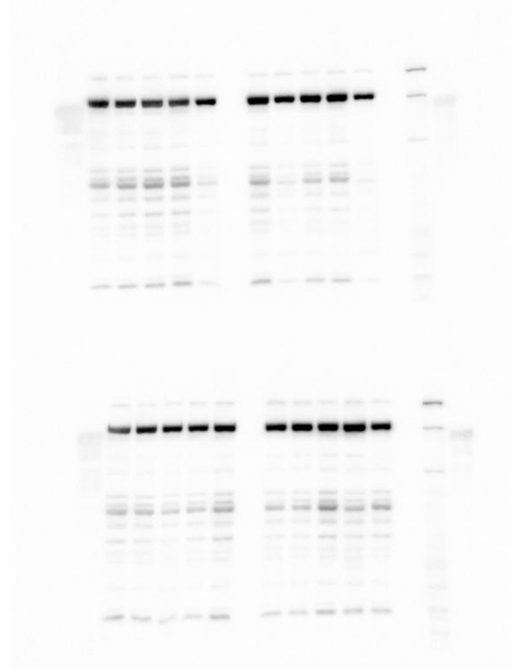

**Figure 4D**<br>Acetyl Lysine Gel (3 and 4)	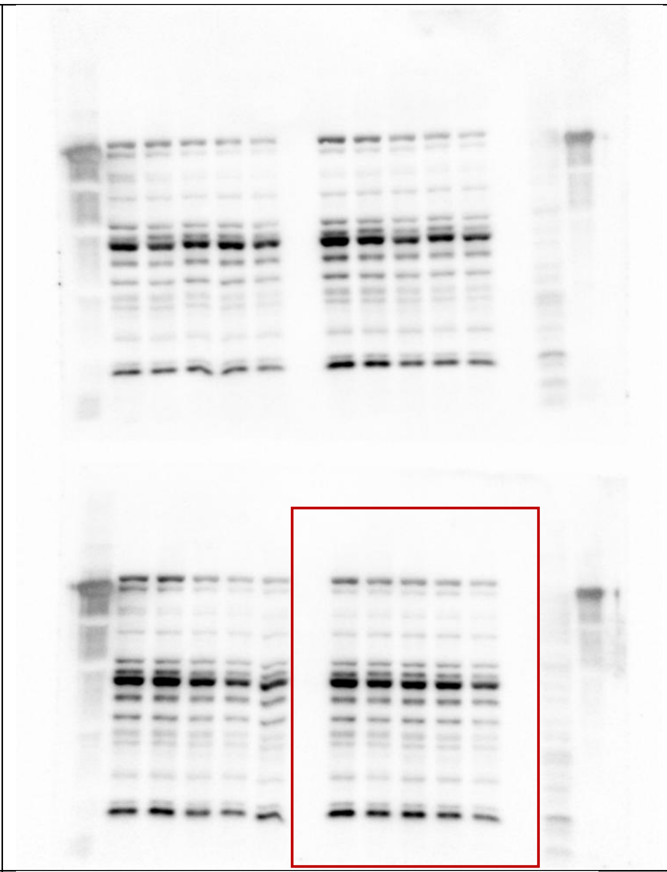
**Figure 4D**<br>Vinculin for Acetyl lysine gel 3 and 4	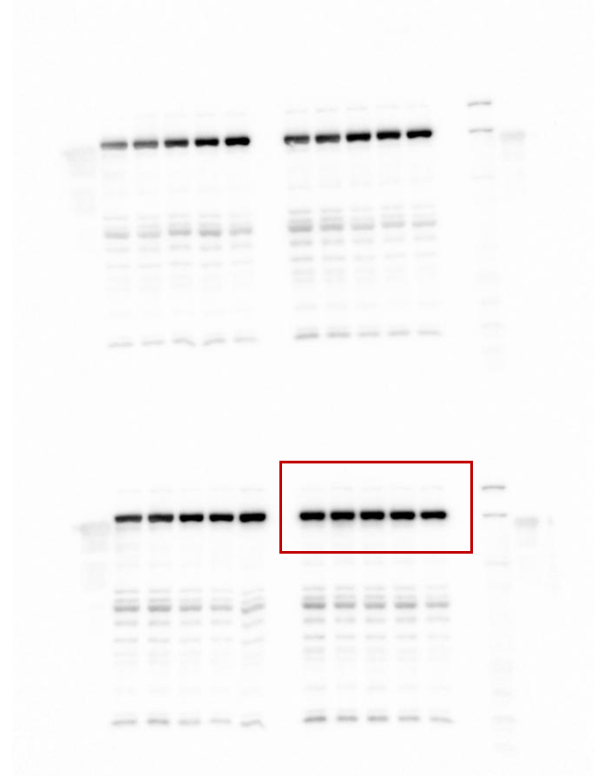
